# The suppressing effects of BTG3 expression on aggressive behaviors and phenotypes of colorectal cancer: An *in vitro* and *vivo* study

**DOI:** 10.18632/oncotarget.15438

**Published:** 2017-02-17

**Authors:** Hua-Chuan Zheng, Hao-Yu He, Ji-Cheng Wu, Jing Li, Shuang Zhao, Gui-Feng Zhao, Hua-Mao Jiang, Xue-Wen Yu, Zhi-Jie Li

**Affiliations:** ^1^ Department of Experimental Oncology and Animal Center, Shengjing Hospital of China Medical University, Shenyang 110004, China; ^2^ Jinzhou Medical University, Jinzhou 121001, China

**Keywords:** colorectal cancer, BTG3, pathobiological behaviors, aggressive phenotypes, gene therapy

## Abstract

Here, we found that down-regulated expression of BTG3 might be positively correlated with colorectal carcinogenesis and its overexpression suppressed proliferation, glycolysis, mitochondrial respiration, cell cycle progression, migration, and invasion, and induced apoptosis, senescence and differentiation in SW480 and SW620 cells. After treated with cisplatin, MG132, paclitaxel and SAHA, *BTG3* transfectants exhibited lower viability and higher apoptosis than the control in both time- and dose-dependent manners. BTG3 overexpression up- regulated the protein expression of Cyclin E, p16, p27, NF-κB, p38α/β, XIAP, Bcl-2, ATG14 and p53, but down-regulated the mRNA expression of *MRP1*, *BCRP*, and *mTOR* in SW480 and SW620 cells. BTG3 overexpression inhibited tumor growth of SW620 cells by suppressing proliferation and inducing apoptosis. It was suggested that down-regulated BTG3 expression might be considered as a marker for colorectal carcinogenesis. BTG3 overexpression might reverse the aggressive phenotypes and be employed as a potential target for gene therapy of colorectal cancer.

## INTRODUCTION

Colorectal cancer (CRC) is one of the most common cancers in the world and accounts for nearly one-tenth of new cases of all cancers. Although the incidence and mortality have been decreasing in the past two decades due to the alteration in risk factors and colorectal cancer screening, it challenges the health of the human being [1, 2]. Therefore, it is essential to improve the diagnosis, treatment and prevention *via* the identification of novel biomarkers and treatment targets.

*BTG3* is distributed to human chromosome 21q21.1 and transcriptionally produces two variants with 132-nucleotide deletion by alternative splicing. Its encoding protein is found to inhibit cell proliferation, cell cycle progression and induce differentiation as a tumor suppressor [3, 4]. BTG3 can interact with E2F1 to suppress the binding of the E2F1-DP1 complex to DNA duplex, resulting in cellular S-phase arrest [5]. BTG3 also interacts with Smad8 receptor- regulated Smad transcription factor [6], and with src to suppress Ras/MAP kinase signaling [7]. BTG3 can be phosphorylated and activated *via* the interaction with checkpoint kinase 1 (CHK1). BTG3-mediated maintenance of genomic stability requires its Lys63 ubiquitination and CHK1 activation [5, 8]. *BTG3* abrogation might enhance bone morphogenetic protein- induced ectopic bone formation [6] and induce lung carcinogenesis [9]. The down-regulated *BTG3* expression was attributable to its promoter methylation in breast, lung, prostate or renal, hepatocellular, and gastric cancer tissues or cells [10–15]. The down- regulated BTG3 expression was also found during ovarian carcinogenesis, and positively correlated with the dedifferentiation, FIGO staging, and adverse prognosis of cancers [16]. Yanagida et al. [17] showed that *BTG3* knockdown promoted proliferation and tumorigenicity of ovarian clear cell carcinoma. Our group also demonstrated that BTG3 overexpression inhibited proliferation, migration, invasion and tumor growth, induced S/G_2_ arrest, differentiation, autophagy, apoptosis and chemosensitivity of gastric cancer cells [18].

To clarify the roles of BTG3 in colorectal carcinogenesis and subsequent progression, we investigated the effects of BTG3 overexpression on cell proliferation, glucose metabolism, apoptosis, senescence, differentiation, invasion and migration of CRC cells and screened the expression of the phenotype-related genes. Moreover, we examined the expression of *BTG3* mRNA and protein in CRCs, and compared them with clinicopathological parameters of cancers. Its promoter methylation was measured in colorectal cancer cells and tissues. Finally, the *in vivo* effect of BTG3 overexpression on tumor growth was determined in nude mice bearing CRC cells.

## RESULTS

### The effects of BTG3 expression in the phenotypes and relevant molecules of CRC cells

**Figure 1:**
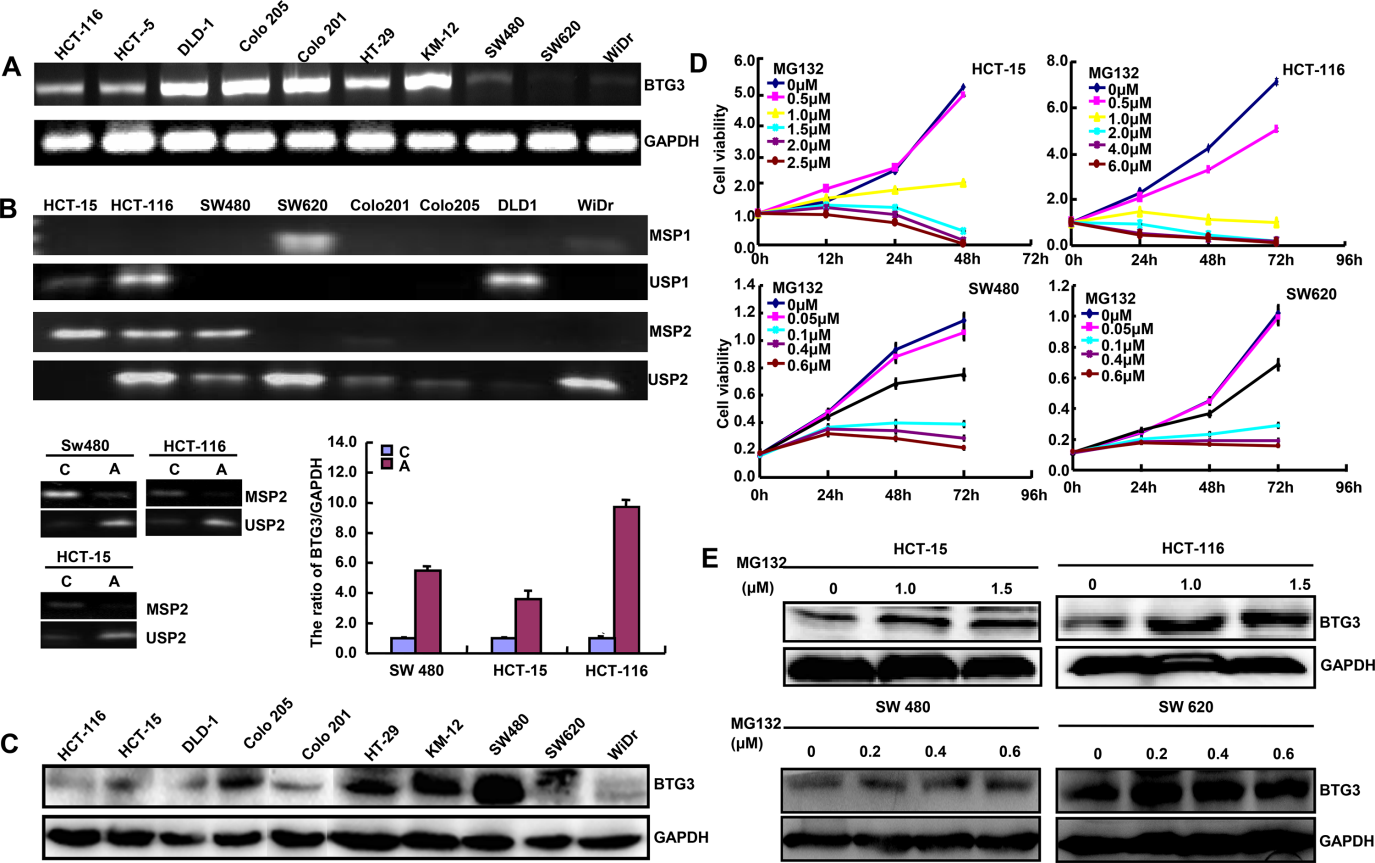
BTG3 expression in colorectal cancer cells *BTG3* mRNA was detected at different levels in colorectal cancer cells with an internal control of *GAPDH* **A**. The promoter methylation of BTG3 was screened by methylation-specific PCR (MSP, B). After treated with 5-Aza-Dc, SW480, HCT-15, and HCT-116 cells showed a lower methylation and a higher expression of *BTG3* mRNA by RT-PCR than the control **B**. The protein was loaded and probed by the anti-BTG3 (29kDa) antibody with GAPDH (42 kDa) as an internal control **C**. After treated with MG132, HCT-15, HCT-116, SW480 and SW620 cells showed a gradually decreased proliferation in either dose- or time-dependent manner **D**. Additionally, BTG3 expression became higher in the MG132-treated four cells than the control, evidenced by Western blot **E**.

We observed a comparatively higher expression of *BTG3* mRNA in DLD-1, Colo 201, Colo 205, and KM-12 (Figure 1A). Its promoter methylation was detected in HCT-15, HCT-116, SW480 and SW620 cells (Figure 1B). After treated with 5-Aza, promoter methylation level of *BTG3* was weakened, but versa for its mRNA expression in SW480, HCT-15 and HCT-116 cells (Figure 1B, p<0.05). However, BTG3 protein expression was higher in Colo 205, HT-29, KM-12 and SW480 (Figure 1C). After treated with MG132, HCT-15, HCT-116, SW480 and SW620 cells showed a gradually decreased proliferation in either dose- or time-dependent manner (Figure 1D, p<0.05) with an increased BTG3 expression (Figure 1E).

**Figure 2:**
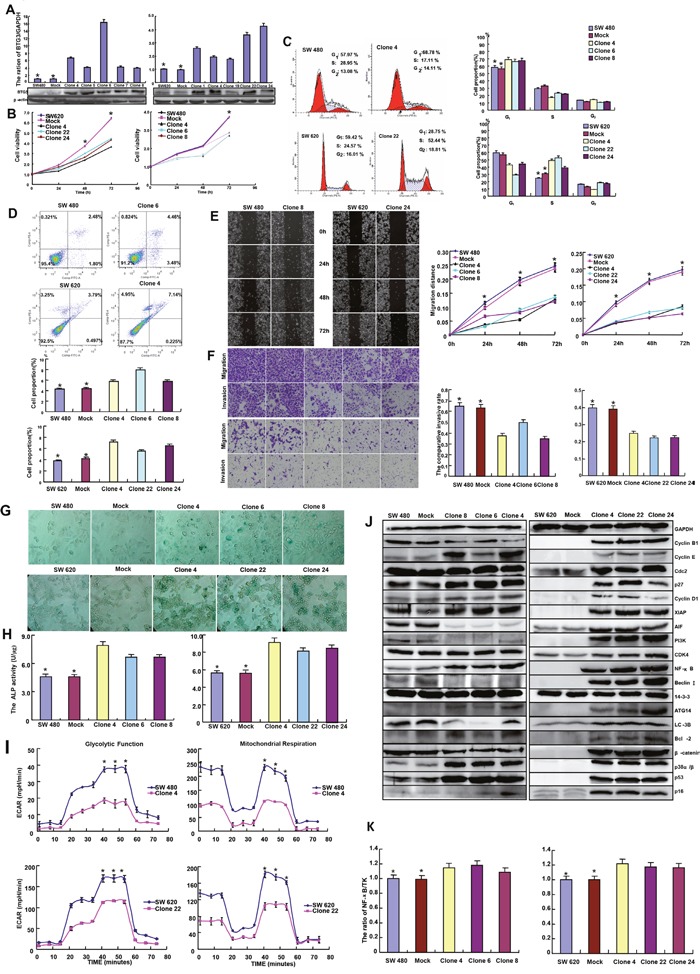
The effects of BTG3 overexpression on the phenotypes and their relevant molecules of colorectal cancer cells After the transfection of pcDNA3.1-*BTG3*, its expression became strong in SW480 and SW620 cells by RT-PCR and Western blot **A**. The transfectants showed a low growth **B**. and G_1_ or S arrest **C**. in comparison with the control and mock. There was an apoptosis-induced effect of BTG3 overexpression in both transfectants, evidenced by Annexin V assay **D**. BTG3-overexpressing cells had a weaker ability to migrate and invade by wound healing **E**. and transwell assays **F**. *BTG3* transfectants showed a higher senescence, a better differentiation, a lower level of glycolysis or mitochondrial function than the control and mock, evidenced by β-galactosidase staining **G**., ALP activity **H**., and metabolism assay **I**. respectively. The expression of phenotype- related molecules was screened by Western blot **J**. There was a high activity of NF-κB promoters in *BTG3* transfectants, compared with the control and mock **K**. Results are representative of 3 different experiments, and data are expressed as mean ± standard deviation. Note: NC, negative control; * p < 0.05, compared with *BTG3* transfectants.

Here, we successfully transfected *BTG3*-expressing plasmid into SW480 and SW620 cells, evidenced by real-time RT-PCR and Western blot (Figure 2A). Both the transfectants showed a lower growth, evidenced by CCK-8 (Figure 2B, p<0.05) and a higher apoptosis by Annexin V-FITC staining in *BTG3* transfectants than the control and mock (Figure 2D, p<0.05). There appeared G_1_ arrest in BTG 3-overxpressing SW480 cells in comparison to the control and mock by PI staining, while S arrest in SW620 cells (Figure 2C). BTG3 overexpression inhibited the migration and invasion of CRC cells according to wound healing (Figure 2E, p<0.05) and transwell chamber assays (Figure 2F, p<0.05), but induced senescence and differentiation, evidenced by β-galactosidase staining (Figure 2G) and alkaline phosphatase (ALP) activity (Figure 2H, p<0.05) respectively. BTG3-overexpressing CRC cells displayed a lower glycolysis and mitochondrial respiration than the mock and control according to oxygen consumption and extracellular acidification rates (Figure 2I, p<0.05).

**Figure 3:**
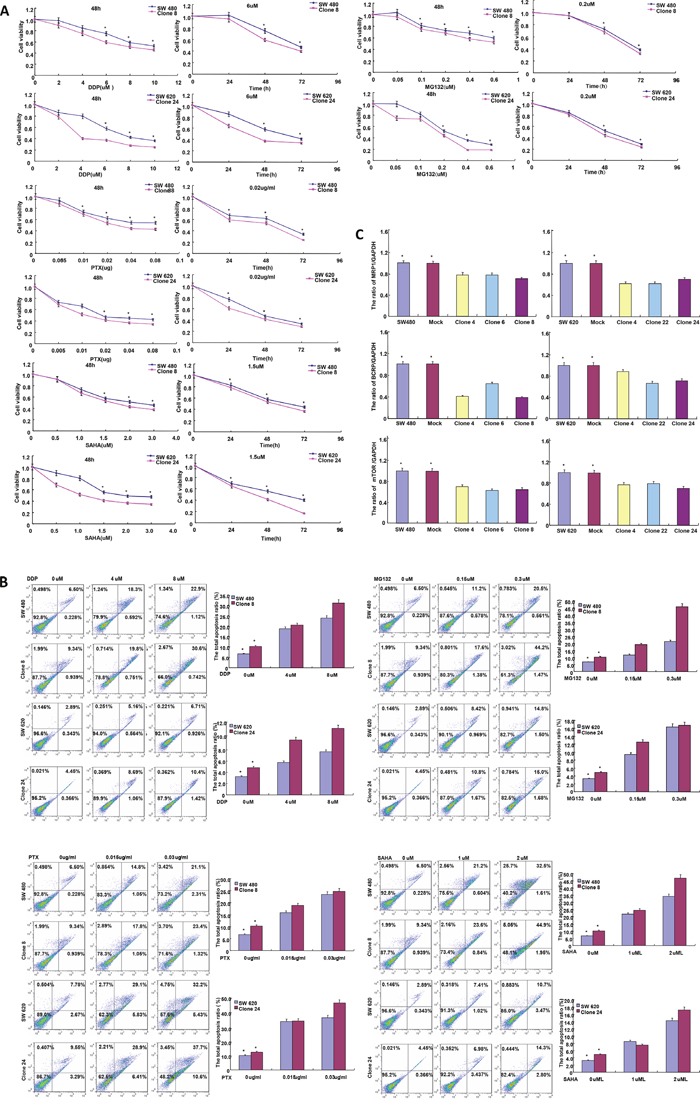
BTG3 expression enhances the sensitivity of colorectal cancer cells to chemotherapeutic drugs After the exposure to cisplatin (DDP), MG132, paclitaxel (PTX), and SAHA, *BTG3* transfectants showed a lower viability and a higher apoptotic level than the control in both concentration- and time-dependent manners **A** and **B**. The chemoresistance-related genes were screened by real-time RT-PCR **C**. * p< 0.05, compared with *BTG3* transfectants.

As shown in Figure 2J, BTG3 overexpression increased the expression of Cyclin E, Cyclin D1, p16, p27, NF-κB, p38α/β, XIAP, Bcl-2, ATG14, and p53 in SW480 and SW620 cells. There was a lower expression of Cyclin B1, AIF, PI_3_K, Beclin 1, LC-3B, and β-catenin in *BTG3* transfectants of SW480 than the mock and control, while the converse was true for SW620. Additionally, transcriptional activity of NF-κB were higher in BTG3 transfectants than the control or mock according to luciferase reporter assay (Figure 2K, p<0.05). After treated with cisplatin, MG132, paclitaxel and SAHA, *BTG3* transfectant exhibited lower viability and higher apoptosis than the control in both time- and dose-dependent manners (Figure 3A-3B, *p*<0.05). BTG3 overexpression decreased the expression of *MRP1*, *BCRP*, and *mTOR* in SW480 and SW620 cells (Figure 3C, *p*<0.05).

### The effects of BTG3 overexpression on the tumor growth of CRC cells in nude mice

**Figure 4:**
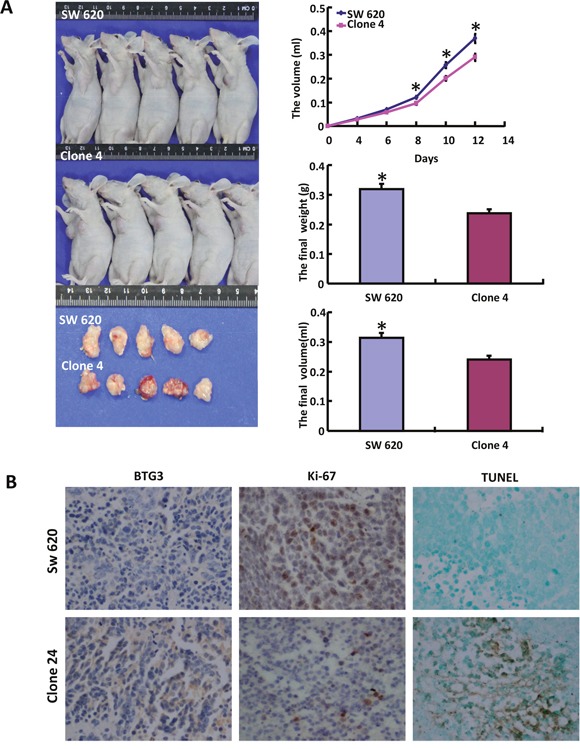
The roles of BTG3 overexpression on the growth of colorectal cancer cells in nude mice The tumor volume and weight were measured by ruling, weighting and capacity measurement **A**. Immunohistochemistry was employed for the detection of BTG3 and ki-67 expression, while TUNEL for apoptotic signal **B**.

SW620 and its *BTG3* transfectant were subcutaneously transplanted into immune-deficient nude mice. As shown in Figure 4A, the tumor volume and weight of both parental cells were larger and heavier than those of *BTG3* transfectants by ruling, weighting and capacity measurement respectively (p<0.05). BTG3 protein was strongly expressed in the cytoplasm of the transfectant, compared with the control. The transfectants showed a low expression of ki-67 (a marker for proliferation) and a strong signal of TUNEL (a marker for apoptosis) in comparison to the control (Figure 4B).

### BTG3 expression in CRC tissues

**Figure 5:**
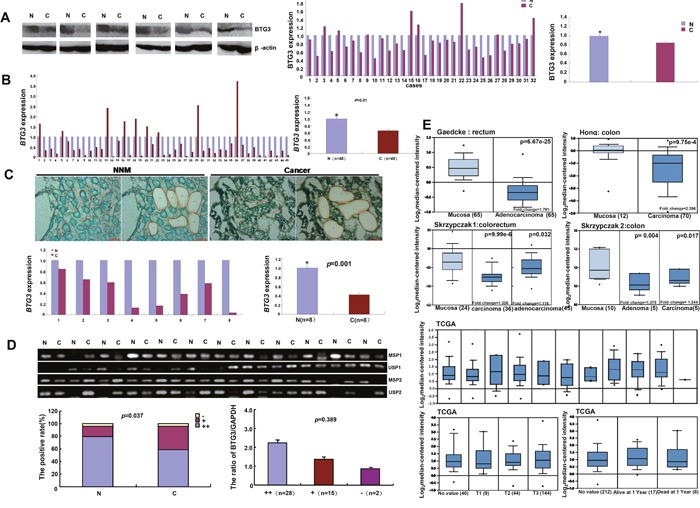
BTG3 expression in colorectal cancer There was less BTG3 expression in the tissue lysates of colorectal cancer than paired mucosa with β-actin as an internal control (p < 0.05, **A**.) by Western blot and densometric analysis. *BTG3* cDNA was amplified by real-time RT-PCR with *GAPDH* as an internal control. A lower *BTG3* mRNA expression was detected in colorectal cancer than matched mucosa (p < 0.05, **B**.), even followed laser capture dissection (p<0.05, **C**.). The higher level of *BTG*3 methylation was detected in normal mucosa than paired cancer, but no relationship was found between the mRNA expression and promoter methylation of *BTG3* in colorectal cancers **D**. Gaedcke's, Hong's, Skrzypczak's, and TCGA's datasets were employed for bioinformatical analysis to compare *BTG3* mRNA expression with the carcinogenesis, TNM staging and prognosis of colorectal cancer **E**. Note: M, methylated; U, unmethylated; N, non-neoplastic mucosa; C, cancer; NC, negative control; -, negative for methylaiton; +, only one site for methylation; ++, two sites for mehtylation; p > 0.05. Data was expressed as mean ±standard error.

BTG3 protein was more detected in colorectal non-neoplastic mucosa (NNM) than that in cancer (Figure 5A, p<0.05). The decreased *BTG3* mRNA expression was seen in CRC, in comparison with paired NNM (Figure 5B, p<0.05). It was also verified by laser capture microdissection (LCM) and real-time RT-PCR (Figure 5C, p<0.05). The promoter methylation of *BTG3* was less frequently detected in CRC than paired NNM (Figure 5D, p<0.05). There was no correlation between mRNA expression and promoter methylation of *BTG3* in CRCs (p>0.05). Then, we used Gaedcke's, Hong's, Skrzypczak's and TCGA's dataset to perform bioinformatical analysis and found that *BTG3* mRNA expression was higher in colorectal normal mucosa than adenoma or cancer (Figure 5E, p<0.05).

**Figure 6:**
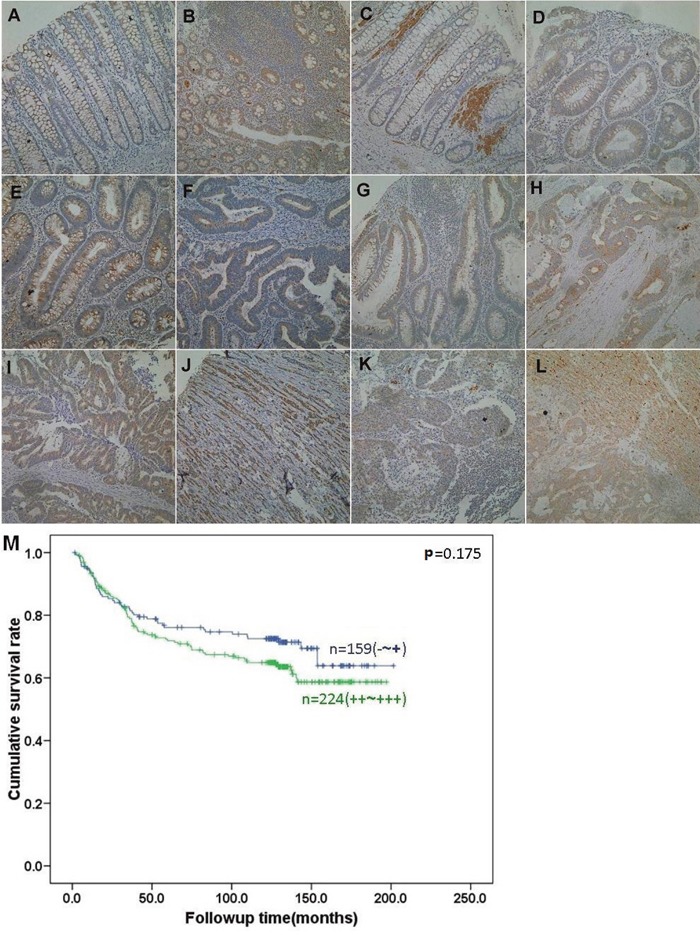
BTG3 expression and its prognostic significance in colorectal cancer BTG3 protein was positively detected in the cytoplasm of colorectal epithelium **A, B**., infiltrating inflammatory cells (A), lymphoid follicle (B), macrophages **C**., adenoma **D, E**., well- differentiated **F, G**., moderately-differentiated **H, I**., and poorly-differentiated **J**. adenocarcinoma, metastatic cancers in lymph node **K**. and liver **L**., and hepatocytes (L). Kaplan-Meier analysis showed no relationship between BTG3 expression status and the cumulative survival of patients with colorectal cancer **M**.

BTG3 protein was distributed in the cytoplasm of colorectal epithelium, infiltrating inflammatory cells, macrophages, hepatocytes, lymphoid follicle, adenoma, and cancers (Figure 6A-6L). BTG3 expression was detectable in colorectal NNM (46.3%, 220/475), adenoma (57.9%, 73/126), primary cancer (82.0%, 396/484), and metastatic cancer in lymph node (80.0%, 120/150) and liver (81.0%, 17/21) respectively. BTG3 expression was statistically higher in colorectal cancer and adenoma than adjacent non-neoplastic mucosa (p<0.05, Table 1 ).

**Table 1:** BTG3 expression in colorectal carcinogenesis and subsequent progression

Groups	n	BTG3 expression				
		-	+	++	+++	PR (%)
Non-neoplastic mucosa	475	255	91	88	41	46.3
Adenoma	126	53	27	30	16	57.9*
Primary cancer	484	88	101	136	159	82.0*
Metastatic cancer in lymph node	150	30	36	52	32	80.0
Metastatic cancer in liver	21	4	5	7	5	81.0

* compared with non-neoplastic mucosa, p<0.001

PR, positive rate

### The relationship between BTG3 expression and clinicopathological parameters of CRC

BTG3 expression was positively correlated with depth of invasion and differentiation of CRCs (Table 2, p<0.05). Follow-up information was available on 383 CRC patients for periods ranging from 2 days to 10.8 years (median=68.6 months). Kaplan-Meier analysis indicated no difference between the cumulative survival rates of patients and BTG3 expression, even stratified by depth of invasion (Figure 6M, p>0.05). Cox’ s proportional hazard model indicated that depth of invasion and distant metastasis were independent prognostic factors for overall CRCs (Table 3, p<0.05).

**Table 2:** Relationship between BTG3 expression and clinicopathological features of colorectal cancers

Clinicopathological features	n	BTG3 expression					
		-	+	++	+++	PR(%)	p value
Age(years)							0.090
<65	261	53	56	74	78	79.7	
≥65	223	35	45	62	81	84.3	
Sex							0.946
male	281	51	58	81	91	81.9	
Female	203	37	43	55	68	81.8	
Depth of invasion							0.030
T_is_-T_2_	125	28	28	36	33	77.6	
T_3_-T_4_	339	52	70	97	120	84.7	
Lymphatic invasion							0.322
-	196	34	41	54	67	82.7	
+	208	38	47	65	58	81.7	
Venous invasion							0.577
-	193	39	39	57	58	79.8	
+	198	32	45	59	62	83.8	
Lymph node metastasis							0.816
-	255	46	55	67	87	82.0	
+	210	38	42	65	65	81.9	
Liver metastasis							0.549
-	449	81	94	130	144	82.0	
+	28	5	6	5	12	82.1	
Distant metastasis							0.172
-	444	81	94	129	140	81.8	
+	36	6	6	7	17	83.3	
TNM staging							0.861
O-II	244	44	54	65	81	82.0	
III-IV	218	38	42	68	70	82.6	
Differentiation							0.037
Well-differentiated	196	30	37	57	72	84.6	
Moderately-differentiated	232	41	53	63	75	82.3	
Poorly-differentiated	32	8	9	9	6	75.0	

PR, positive rate; T_is_, cancer *in situ*; T_1_, lamina propria and submucosa; T_2_, muscularis propria; T_3_, subserosa and exposure to serosa; T_4_, invade other organs or perforate visceral peritoneum

**Table 3:** Multivariate analysis of clinicopathological variables for the survival of the patients with colorectal cancer

Clinicopathological parameters	Relative risk ( 95%CI)	p value
Age(≥65years)	0.884(0.561-1.395)	0.597
Sex(Female)	0.817(0.520-1.283)	0.380
Depth of invasion (T_2-4_)	5.873(2.058-16.762)	0.001
Lymphatic invasion(+)	1.269(0.756-2.129)	0.367
Venous invasion(+)	1.088(0.646-1.830)	0.752
Lymph node metastasis(+)	1.638(0.482-5.568)	0.429
Liver metastasis	0.357(0.071-1.793)	0.211
Distant metastasis	11.330(2.466-52.057)	0.002
TNM staging(I-II)	1.746(0.453-6.729)	0.418
Differentiation (moderately and poorly)	1.131(0.792-1.616)	0.497
BTG3 expression (++~+++)	1.227(0.775-1.942)	0.384

CI, confidence interval; TNM, tumor node metastasis

## DISCUSSION

In line with the results of oncomine analysis, we found that *BTG3* expression was decreased in CRC, compared with adjacent NNM by Western blot and real-time RT-PCR, even followed by LCM. These findings suggested that down-regulated BTG3 expression contributed to the malignant transformation of colorectal epithelial cells, consistent with these documents [14, 15, 18, 19]. As reported previously [18], only *BTG3b* mRNA was found in colorectal cancer and NNM although *BTG3*a and *3b* were detectable in lung cancer cells and tissues [15]. After the exposure to 5-Aza, CRC cells showed the attenuated promoter methylation and strengthened *BTG3* mRNA expression, which was reported in gastric, breast, renal and prostate cancer cells [10–12, 18]. In combination with no correlation between *BTG3* methylation and mRNA expression in CRC, it was speculated that *BTG3* methylation was partially responsible for its silenced expression. In contrast, more frequent methylation of BTG3 was found in colorectal mucosa than cancer, similar to its mRNA, while versa for BTG3 immunopositivity. The paradoxical phenomenon might be hypothesized to correlate with *BTG3* methylation, weaker BTG3 expression in cytosol-marginal stromal cells, and BTG3 degradation by ubiquitin-proteasome system [20] as demonstrated in the present study.

Here, BTG3 overexpression enhanced ALP level of CRC cells, supporting the positive link between BTG3 overexpression and differentiation of CRCs. There appeared BTG3 overexpression in deeply-invasive cancer, while BTG3 overexpression suppressed proliferation, migration, invasion, tumor growth, glycolysis and mitochondrial respiration with the induction of apoptosis or senescence as the reports about gastric cancer [18], and esophageal adenocarcinoma [19] cells. Ectopic BTG3 expression reversed the aggressive behaviors of gastric cancer and hepatocellular cancer cells *in vitro* [14], and inhibited the tumor growth of lung and gastric cancers *in vivo* [15, 18]. In contrast, Provenzani et al [21] found that *BTG3* mRNA had about 1.83 fold higher in primary than metastatic cancer of colon by cDNA microarray. Exogenous BTG3 protein suppresses the levels of MMP-2 and PAI-1 expression in lung cancer cells [9]. Reportedly, BTG3 expression was inversely correlated with differentiation, lymph node metastasis, or distant metastasis of esophageal, gastric and hepatocellular cancers [14, 18, 19]. BTG3 binds and suppresses AKT so as to decrease β-catenin/TCF activity, down-regulate mesenchymal markers, and consequently reduce cell migration and tumor growth [22]. Another report showed that BTG3 overexpression was found to inhibit cell growth, invasion, metastasis and epithelial–mesenchymal transition (EMT), induced cell cycle arrest of SW480 cells *via* Wnt/β-catenin signaling pathway [23]. Therefore, we concluded that BTG3 overexpression might be a feedback reaction and suppress aggressive phenotypes so that it might be employed as a potential target of gene therapy for CRCs.

Furthermore, *BTG3* transfectant showed a higher chemosensitivity to cisplatin, MG132, paclitaxel and SAHA, which was positively correlated with a high apoptotic induction. Additionally, BTG3 overexpression results in the mRNA hypoexpression of chemoresistance- related genes, such as *MRP1*, *BCRP*, and *mTOR*. MDR1 and BCRP belong to efflux transporters of the ATP-binding cassette family and pumps many foreign substances out of cells [24]. Chemoresistance is induced or acquired through the activation of the PI_3_K /Akt/mTOR pathway in some malignancies [25]. Taken together, BTG3-mediated chemosensitivity was positively linked to the down-regulated expression of the chemoresistance-related genes. However, the detailed mechanisms would be investigated in the future.

Although both SW480 and SW620 cells are separated from primary and lymph node metastasis foci of the same CRC patients, the inconsistent expression of Cyclin B1, AIF, PI_3_K, Beclin 1, LC-3B, and β-catenin in BTG3 might underlie the molecular mechanisms of the differences in the biological behaviors of SW480 and SW620, even their BTG3 transfectants. In apoptosis, Bcl-2 can interact with Bax on the mitochondrial membrane to suppress the apoptosis [26]. XIAP might function as apoptotic inhibitor by binding to and suppressing caspase activation [27]. The up-regulated expression of both proteins in *BTG3* transfectants was demonstrated to not correlate with BTG3-induced apoptosis in CRC cells. According to LC-3B expression, the effects of BTG3 on autophagy were different between SW420 and SW680, and positively linked to Beclin 1 expression, indicating that BTG3-induced autophagy was dependent on Beclin-1 and belonged to canonical pathway. Here, BTG3 overexpression was found to activate NF- κB pathway by up-regulating its transcriptional activity and expression, but its biological effects should be deeply investigated.

Although there *in vivo* and *vitro* appeared a low proliferation in *BTG3* transfecants, BTG3 overexpression resulted in G_1_ arrest in SW480 cells and S arrest in SW620 cells. Reportedly, Cyclin E and D1 activate Cdks and induce the transition between G_1_ and S phase [28]. Cyclin B1-Cdk1 is involved in the early events of mitosis and Cdc25B activates Cdc2 for the entry into mitosis [29]. Therefore, the higher expression of Cyclin D1, Cyclin E, cdc2 and cdk4 were responsible for S arrest in BTG3-overxpressing SW620. Overexpressed p16 and p27 bind to cyclins and cdks, which cause G_1_ arrest [28, 30]. Activated p53 binds DNA and activates expression of p21 to inhibit G_1_/S transition. BTG3-mediated cell cycle arrest might be due to the overexpression of above-mentioned 3 proteins.

BTG3 expression was found to positively associate with favorable prognosis of gastric and ovarian cancers [13, 16]. Lv et al. [14] found that the hepatocellular cancer patients with lower BTG3 expression had shorter overall survival time than the ones with higher BTG3 expression. In contrast, no link between BTG3 expression and the survival time of the patients with CRC was demonstrated even though stratified according to depth of invasion, in line with our report of gastric cancer [18]. Multivariate analysis demonstrated that depth of invasion and distant metastasis were independent prognostic factors for overall CRCs. In our another report, a positive link between BTG1 expression and poor survival of gastric cancer patients was found, which depended upon invasive depth of the cancers [31]. These findings suggested that BTG3 expression could not be employed to indicate the prognosis of CRC patients.

In summary, down-regulated BTG3 expression might be positively correlated with the malignant transformation of colorectal epithelial cells and should be considered as a good biomarker for colorectal carcinogenesis. Promoter methylation of *BTG*3 is partially responsible for its down- regulated expression. BTG3 overexpression might *in vitro* and *vivo* reverse the aggressive phenotypes of CRC cells and be employed as a target molecule for gene therapy.

## MATERIALS AND METHODS

### Cell culture and transfection

CRC cell lines were kindly presented by Prof. Miyagi, Clinical Research Institute, Kanagawa Cancer Center, Japan. They were cultured in RPMI 1640 (Colo201, Colo205, DLD-1. HCT-15, HCT-116, HT-29, KM-12, SW480 and SW620) and DMEM (WiDr) medium added with 10% FBS, 100 units/mL penicillin, and 100 μg/mL streptomycin in an atmosphere of 5% CO_2_ at 37°C. SW480 and SW620 cells transfected with pcDNA3.1-*BTG3* or pcDNA3.1 vector, and selected by G418 according to the manufacturer's instructions (QIAGEN, USA). We treated CRC cells with 5-aza-20-deoxycytidine (5-Aza-dC, DNA demethylating agent), cisplatin (a platinum- containing DNA crosslinker), MG132 (a proteasome inhibitor), paclitaxel (a mitotic inhibitor), and SAHA (a histone deacetylase inhibitor). All cells were pelleted by centrifugation, rinsed with PBS, and subjected to protein and RNA extraction.

### Proliferation assay

Cell counting Kit-8 (CCK-8, Japan) was used to measure cell viability. Briefly, 4000cells/well were seeded on 96-well plate, which was added with 10 μL CCK-8 solution at different time points. After the incubation for 3 h, optical density was read without cover at 450 nm.

### Cell cycle analysis

The cells were harvested, washed by PBS and fixed in cold ethanol for 4 h at −20°C. After washed by PBS, the cells were immersed with 1mL RNase (0.25 mg/mL) at 37°C for 1 h. The cells were pelleted, resuspended in propidium iodide (PI, 50μg/mL) and incubated in the dark for 30 min. Finally, flow cytometry was carried out to detect PI signal.

### Apoptosis assay

Flow cytometry was carried out with PI and FITC-labeled Annexin V (*KeyGEN* Biotech) to examine apoptosis as the instructions recommend.

### Wound healing assay

1.0×10^6^ cells/well was seeded in 6-well culture plates, and scraped with a 100uL yellow pipette tip to create a scratch when they had grown to confluence at 80%. After that, cells were washed by PBS and grown in FBS-free medium. Cells were photographed and the scratch area was calculated at the different time points using Image J software.

### Cell invasion assays

For invasive assay, 2.5 × 10^5^ cells were seeded in the matrigel-coated insert on the top of the chamber with serum-free medium. The low compartment was filled with 10%-FBS medium as a chemoattractant. As a control, migration assay was performed except no matrigel coating. After incubated for 24 h, cells on the membrane were scrubbed, washed with PBS, fixed in 100% methanol and stained with Giemsa dye for photography and measurement. Finally, the invasive rate was calculated as invasive cells/ migrative cells.

### ALP activity

ALP activity was used as a marker of colorectal differentiation. Briefly, cells were harvested, homogenized and subjected to ALP activity and protein assays using Diagnostics ALP reagent (Sigma) and Biorad protein assay kit respectively. ALP activity was calculated as U per μg of protein.

### β-galactosidase staining

β-galactosidase staining kit (Beyotime, Shanghai, China) was employed to indicate the senescence. The protocol was executed as recommended by the kit instruction.

### Metabolism assays

Both oxygen consumption rates and extracellular acidification rates were measured in XF media (nonbuffered RPMI 1640 containing either 10mM or 25 mM glucose or galactose, 2 mM L-glutamine, and 1 mM sodium pyruvate) under basal conditions and in response to mitochondrial inhibitors, 1 mM oligomycin and/or 100 nM rotenone + 1 mM antimycin A (Sigma) on XF-96 Extracellular Flux Analyzers (Seahorse Bioscience).

### Luciferase reporter assay

The luciferase reporter assay was performed using Dual-Luciferase®Reporter Assay System (Promega, USA) as recommended by the manufacturer. NF-κB-mediated gene transcription activity was determined by the ratio of pGL3- NF-κB luciferase activity, which was normalized to Renilla luciferase activity of the control plasmid.

### Subjects

Colorectal cancers (n=484), adenoma (n=126), adjacent NNM (n=475), metastatic foci in lymph node metastasis (n=150) and in liver (n=21) were collected from the surgical resection in the Affiliated Hospital of Kanagawa Cancer Center (Japan) between 1999 and 2008. The patients with CRC were 281 men and 203 women (26~85years, mean=64.1 years). Forty-five cases of CRCs and paired NNM were collected from the Shengjing Hospital of China Medical University (China) and frozen in −80°C until use. None of the patients received chemotherapy, radiotherapy or adjuvant before surgery. All of them provided consent for use of tumor tissues for clinical research. Ethical Committee of our hospital and Kanagawa Cancer Center approved the research protocol. We followed up the patients by consulting their case documents or calling.

### Pathology

All tissues were fixed in 10% neutral formalin and prepared for paraffin-embedded block and 4 μm-thick section. These sections were subjected to hematoxylin-and-eosin staining for routine pathological examination, including tumor size, depth of invasion, lymphatic and venous invasion, lymph node metastasis and liver metastasis. The staging for each colorectal cancer was evaluated according to tumor-node-metastasis system [32]. Histological architecture of CRCs was described in terms of WHO's classification [33].

### Xenograft models

Locally bred female 6-week-old Balb/c nude mice were used for implantation. They were maintained under specific pathogen-free condition. Housing and all procedures were approved by the committee for animal experiments guidelines on animal welfare of our hospital. Xenografts were established by subcutaneous injection of 1× 10^6^ cells per mouse to axilla (n=10 mice /group). For each tumor, measurements were made using calipers, and tumor volumes were calculated as follows: width^2^× length ×0.52. After anesthetization, the mice were photographed, and sacrificed. Tumors was weighted and cupped in PBS for the volume. After that, the part of tumors were fixed in 4% paraformaldehyde, and embedded in paraffin for following experiments.

### LCM and RNA extraction

Colorectal cancer and glandular epithelial cells were dissected from 8-μm-thick and stained tissue slides (Staining Kit, Ambion) by an Arcturus Veritas™ laser capture microdissection system (Life Technologies) for total RNA extract using Pure Link RNA Mini Kit (Ambion).

### RT-PCR

Total RNA was extracted from colorectal cancer cells or tissues using QIAGEN RNeasy mini kit (Germany), and subjected to cDNA synthesis using AMV transcriptase and random primers (Takara, Japan). According to the Genbank, oligonucleotide primers were designed as described previously [18]. General and real-time RT-PCR amplification was performed using Hotstart Taq™ polymerase and SYBR Premix Ex Taq™ II kit (Takara) respectively.

### Methylation-specific PCR (MSP)

We extracted genomic DNA from colorectal cancer cells and tissues using QIAamp DNA Mini kit (QIAGEN). DNA was modified chemically with sodium metabisulphite using EZ DNA Methylation-Gold Kit (ZYMO Research). The bisulfite-modified DNA was amplified by using primer pairs that specifically amplify either methylated or unmethylated *BTG3* as described previously [18].

### Western blot

Denatured protein was separated on an SDS-polyacrylamide gel and transferred to Hybond membrane. The membrane was in turn blocked in 5% skim milk, primary (Table 4) and secondary horseradish peroxidase (DAKO) antibody. Bands were visualized with Fuji4010 image system (GE Bioscience) by ECL-Plus detection reagents (Santa Cruz). Either β-actin or GAPDH was employed as the internal control antibody.

**Table 4:** The antibodies used in the present study

Names	Source	Company
BTG3	Rabbit	Proteintech Biotech. Inc.
Cyclin B1 (GNS1)	Mouse	Santa Cruz Biotech. Inc.
CyclinE (HE12)	Rabbit	Santa Cruz Biotech. Inc.
Cdc2 (B-6)	Mouse	Santa Cruz Biotech. Inc.
p27	Mouse	Santa Cruz Biotechnology
Cyclin D1 (H-295)	Rabbit	Santa Cruz Biotech. Inc.
XIAP(H-202)	Rabbit	Santa Cruz Biotech. Inc.
AIF(E-1)	Mouse	Santa Cruz Biotech. Inc.
PI_3_K	Rabbit	Abcam
Cdk4 (C-22)	Rabbit	Santa Cruz Biotech. Inc.
NF-κB	Rabbit	Cell signaling
Beclin 1	Rabbit	Abcam
14-3-3 (H-8)	Mouse	Santa Cruz Biotechnology
ATG14	Rabbit	Cell signaling
LC-3B	Rabbit	Wanleibio
Bcl-2 (C 21)	Rabbit	Santa Cruz Biotech. Inc.
β-catenin(C-18)	Goat	Santa Cruz Biotech. Inc.
p38α/β(H-147)	Rabbit	Santa Cruz Biotech. Inc.
p53 (FL-393)	Rabbit	Santa Cruz Biotech. Inc.
p16(C-3)	Goat	Santa Cruz Biotech. Inc.
β-actin(C-4)	Mouse	Santa Cruz Biotech. Inc.
GAPDH	Rabbit	Wanleibio

### Tissue microarray (TMA) and immunohistochemistry

Two-mm-in-diameter TMA was prepared using a Tissue Microarrayer (AZUMAYA KIN-1, Japan). TMA was consecutively cut into 4-μm-thick sections, which were deparaffinised, rehydrated, and subjected to antigen retrieval in target retrieval solution (TRS, DAKO).The sections were quenched with 3% hydrogen peroxide and incubated with 5% bovine serum albumin. The sections were incubated with the rabbit antibody against BTG3 (Sigma; 1:100) or ki-67 (DAKO, 1:100), and then treated with the anti-rabbit conjugated to horseradish peroxidase (DAKO, USA) antibody. Binding sites were visualized with 3, 3'-diaminobenzidine. After counterstained with Mayer's hematoxylin, the sections were dehydrated, cleared and mounted. Omission of the primary antibody was used as a negative control.

BTG3 protein was positively localized in the cytoplasm, while ki-67 in nucleus. One hundred cells were randomly selected and counted from 5 representative fields of each section blindly by two independent observers (Zheng HC and Zhao S). The inconsistent data were determined for final agreement by both persons. The expression positivity was graded and counted as follows: 0 =negative; 1 = 1-50%; 2 = 50-74%; 3 ≥75%. The staining intensity score was graded as follows: 1 = weak; 2 = intermediate; and 3 = strong. The scores for BTG3 positivity and staining intensity were multiplied to obtain a final score, which determines their expression as (- = 0; + = 1-2; ++ = 3-5; +++ = 6-9).

### Terminal digoxigenin-labeled dUTP nick-end labeling (TUNEL)

Cell apoptosis was assessed using TUENL, a method that is based on the specific binding O-TdT to the 3-OH ends of DNA. Therefore, ApopTag Plus Peroxidase *In Situ* Apoptosis Detection Kit (Millipore) was used according to the recommendation. Omission of the working strength TdT enzyme was considered as a negative control.

### Oncomine analysis

The individual gene expression level of *BTG3* was analyzed using Oncomine (www.oncomine.org), a cancer microarray database and web-based data mining platform for a new discovery from genome-wide expression analyses. We compared the differences in *BTG3* mRNA level between colorectal mucosa and adenoma/cancer. All data were log-transformed, median centered per array, and standard deviation normalized to one per array.

### Statistical analysis

*Spearman’s* correlation test and student t test were used to analyze the rank data and compare the means respectively. *Kaplan-Meier* survival plots were generated and comparisons between survival curves were made with the log-rank statistics. *Cox*'s proportional hazards model was employed for multivariate analysis. SPSS 10.0 software was applied to analyze all data and p<0.05 was considered statistically significant.
